# Supplementary material for: Contribution of Capillary Zone Electrophoresis Hyphenated with Drift Tube Ion Mobility Mass Spectrometry as a Complementary Tool to Microfluidic Reversed Phase Liquid Chromatography for Antigen Discovery
Source: Int J Mol Sci. 2022 Nov 1;23(21):13350. doi: 10.3390/ijms232113350 (PMC9659090; doi:10.3390/ijms232113350)

Contribution of Capillary Zone Electrophoresis Hyphenated with Drift Tube Ion-mobility Mass Spectrometry as a Complementary Tool to Microfluidic Reversed Phase liquid Chromatography for Antigen Discovery

Marie-Jia Gou[1], Murat Cem Kose[2], Jacques Crommen[1], Cindy Nix[1], Gaël Cobraiville[1], Jo Caers[2,3] and Marianne Fillet[1*]

Table S1. Sequence, m/z ratio and pI of 25 peptides identified using both BFS and PVA capillaries. Peptides are sorted by increasing migration time in BFS capillary.

Sequence	m/z	Charge	Mass (Da)	pI	MT-BFS (min)	MT-PVA (min)
VIEPVKR	280.8469	3	840.53	8.72	25.27	30.8310
NVYIK	318.686	2	636.37	8.59	27.47	32.2300
VEDALHATR	337.8473	3	1011.52	5.32	28.25	32.6420
GYRPQFYFR	411.8769	3	1233.62	9.99	28.63	32.7170
SYPLDIHNVQDHLK	420.4705	4	1678.85	5.93	28.72	33.2677
IGIQPGHIHKPGK	461.2727	3	1381.81	10	29.55	33.4260
FAENAYFIK	551.781	2	1102.56	6	30.54	34.3123
GNFDLEGLER	575.2809	2	1149.55	4.14	31.89	35.0297
AVEIGSFLLGR	581.3341	2	1161.66	6.05	33.16	36.0417
NIFGYQYTIPTHQGR	598.9755	3	1794.89	8.6	33.74	36.4447
DTTTIIDGVGEEAAIQGR	615.9756	3	1845.92	3.91	34.02	37.1473
ELANVQDLTVR	629.3494	2	1257.68	4.37	34.06	37.2237
TTDVTGTIELPEGVEMVMPGDNIK	652.3935	2	1303.78	8.41	34.95	38.0027
AFDQIDNAPEEK	688.8246	2	1376.63	3.91	35.55	38.1320
KYDIPVVMDSAR	697.361	2	1393.71	5.96	35.72	38.8093
LAATIAQLPDQIGAK	755.4356	2	1509.86	5.84	36.65	39.0623
DDSFFDVYTECR	777.317	2	1553.62	3.84	37.07	39.2707
TLCVVQEGFPTYGGLEGGAMER	791.0434	3	2371.11	4.25	37.33	39.6523
LLPHIPADQFPAQALACELYK	799.0847	3	2395.25	5.32	37.76	39.9040
IELSSAQQTDVNLPYITADATGPK	844.7641	3	2535.28	4.03	38.09	41.5890
TTDVTGTIELPEGVEMVMPGDNIK	849.4201	3	2546.23	3.83	39.68	42.2980
LGYNLVVLDSQNNPAK	872.9659	2	1744.92	5.83	39.77	43.0197
FCGAEGLNNVITLSTFR	949.9789	2	1898.94	6	41.11	43.1617
GIEEVGPNNVPYIVATITSNSAGGQPVSLANLK	1103.9138	3	3309.73	4.53	41.16	43.2217
IAQVQYLVDGLEEIGVVCQQAGGHAAFVDAGK	1114.8996	3	3342.68	4.31	43.28	44.2377

Figure S1. Diagram of the CZE-DTIMS-QTOF (a) and RPLC-Chip-DTIMS-QTOF (b) setups used in this study.

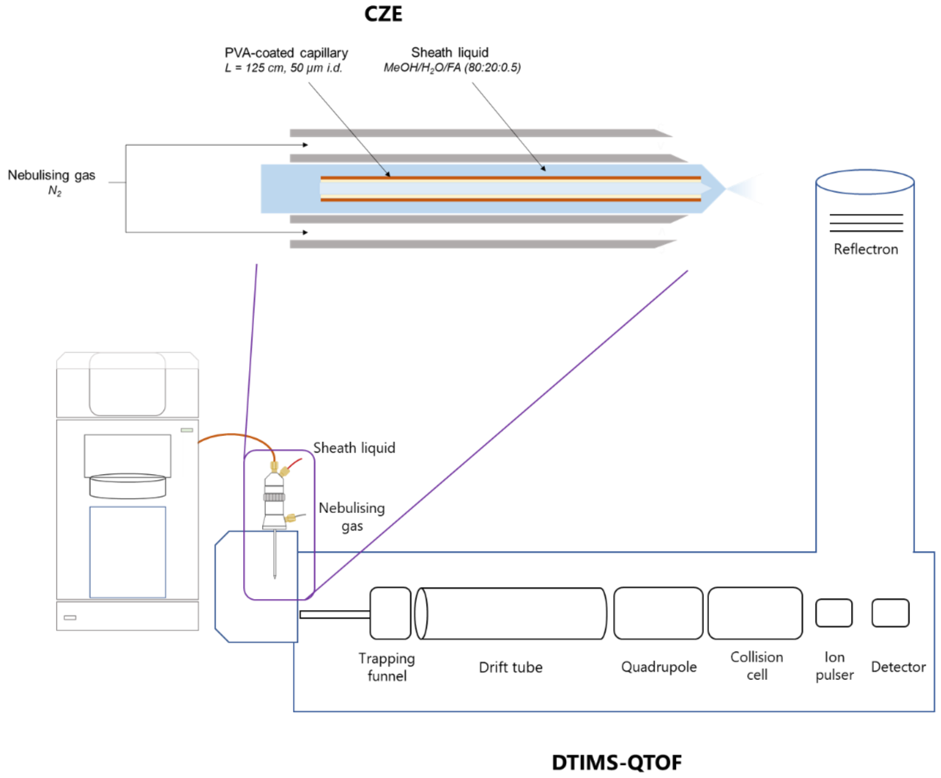

(a)

(b)

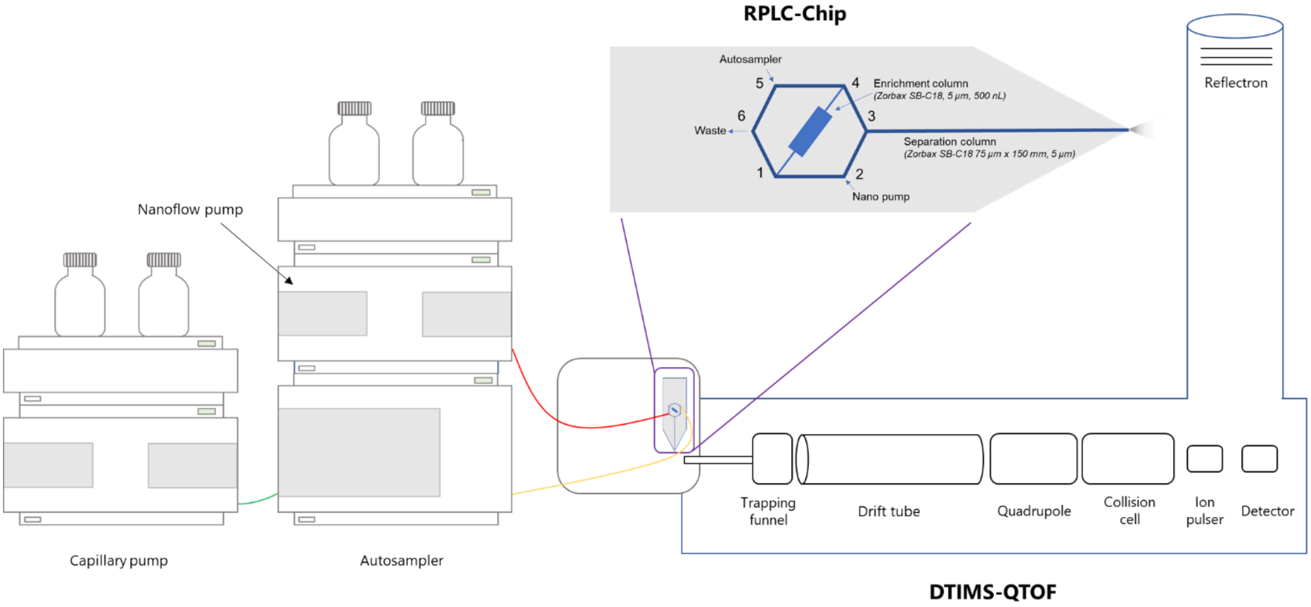

Figure S2. Number of (a) peptides and (b) proteins obtained by analyzing four concentrations of E. coli digest using BFS and PVA-coated capillaries. Statistic comparisons were performed using GraphPad Prism (La Jolla, CA, USA). (Mann-Whitney test; ***, P ≤ 0.001 and ****, P ≤ 0.0001)

(a)

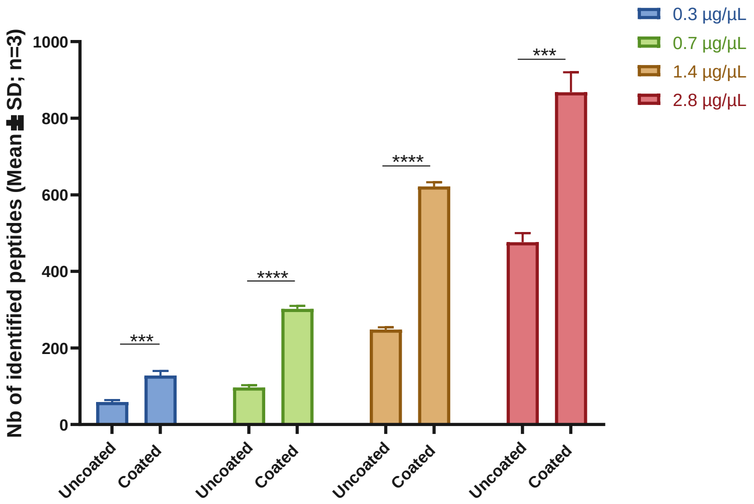

(b)

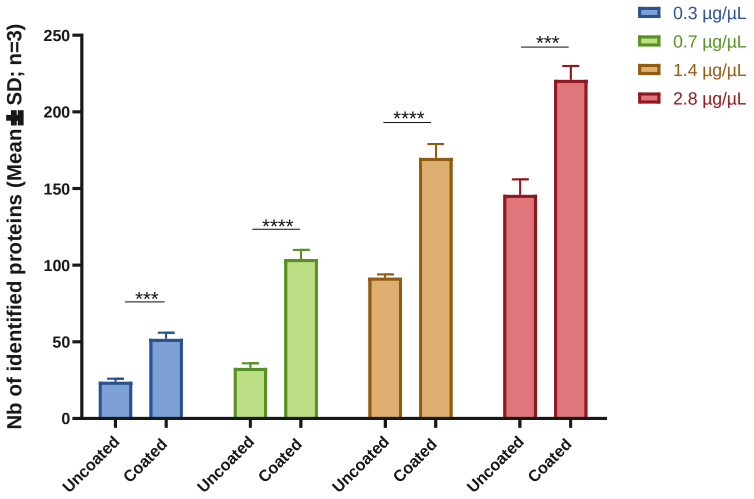

Figure S3. Total ion count (TIC) of 0.25 µg of E. coli digest injected in as BFS capillary (top) and a PVA-coated capillary (down).

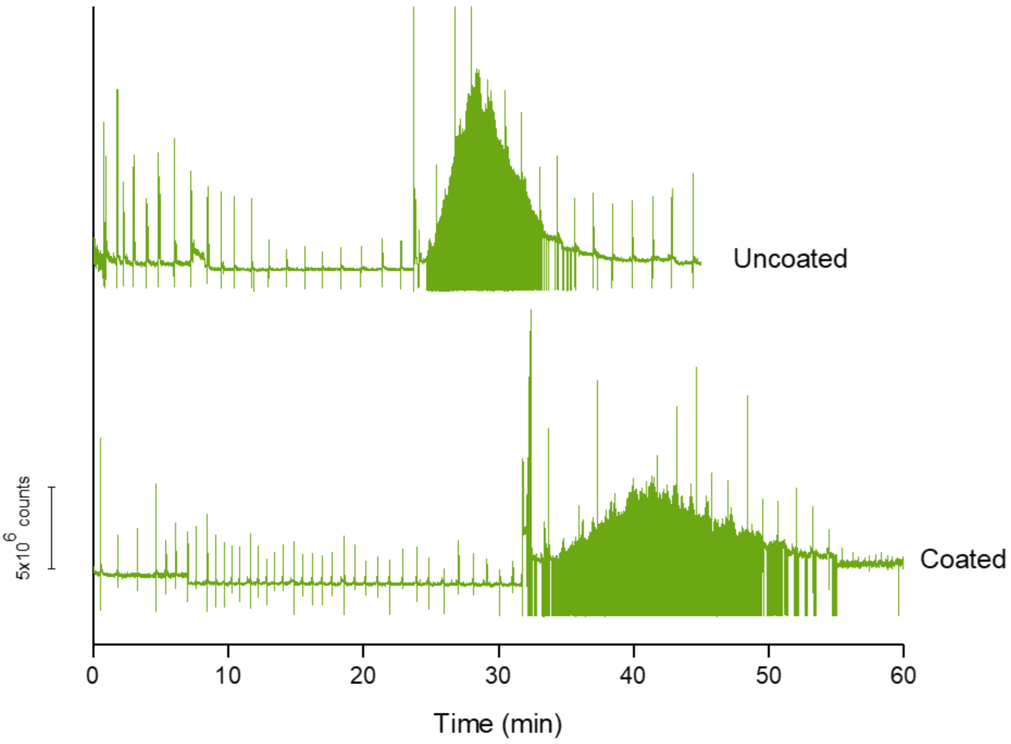

Figure S4. Comparison of peak capacities obtained with triplicate injections of E. Coli digest using BFS and PVA-coated capillaries. Statistic comparisons were performed using GraphPad Prism (La Jolla, CA, USA). (Mann-Whitney test; *, P ≤ 0.05)

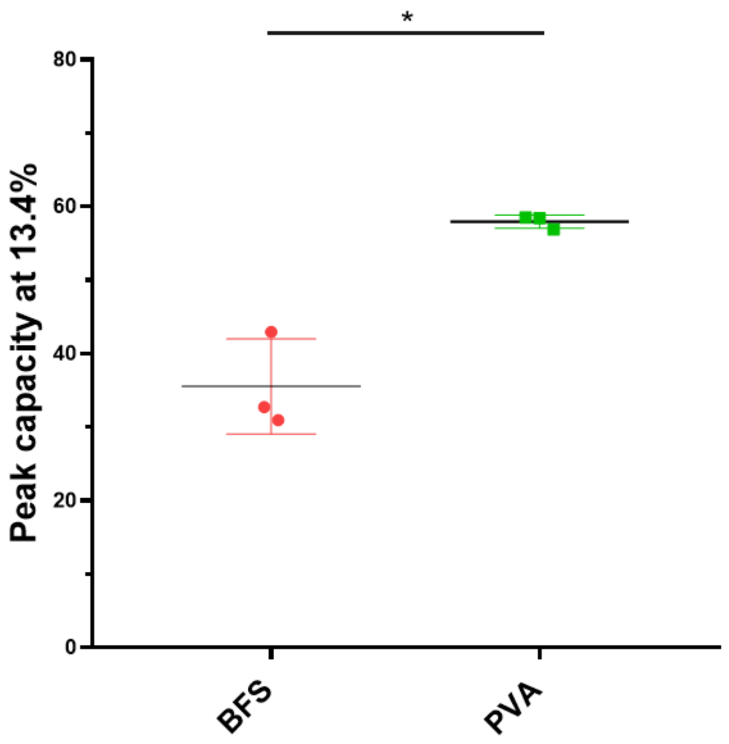

Figure S5. Influence of injected sample volume on the number of identified proteins using four different concentrations of E. coli digest. Statistic comparisons were performed using GraphPad Prism (La Jolla, CA, USA). (Mann-Whitney test; *, P ≤ 0.05 and **, P ≤ 0.01)

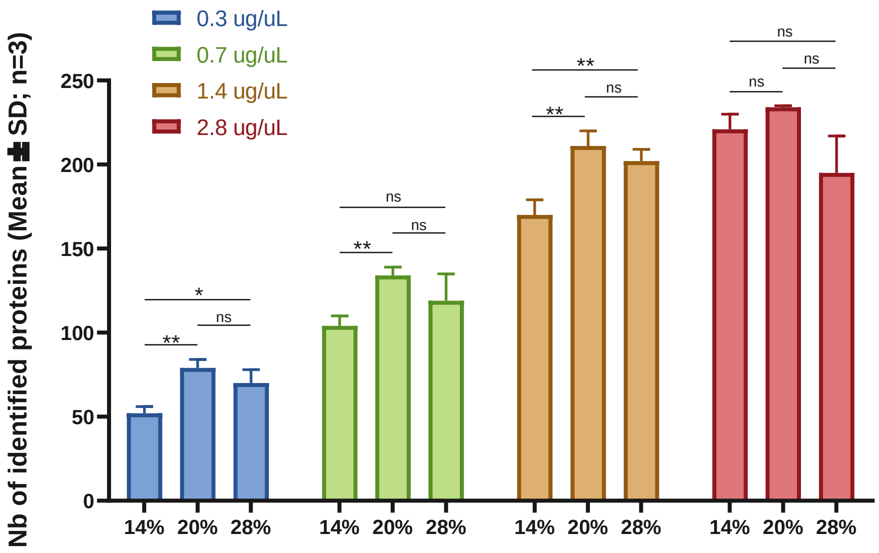

<u>Figure S6.</u> Uniqueness of identified proteins by analyzing LP-1 cell lines using RPLC-Chip and CZE coupled with DTIMS-QTOF.

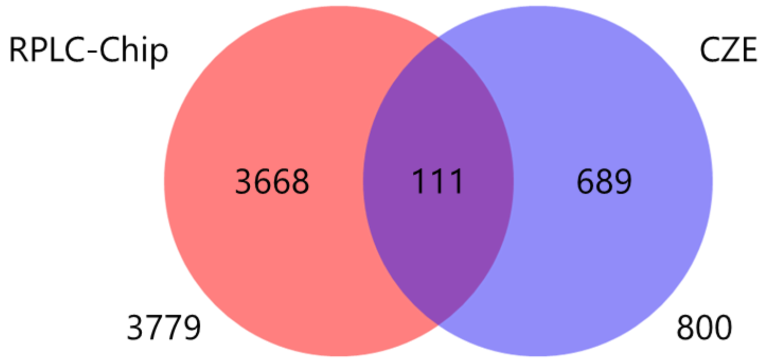

Figure S7. Univariate comparison of various physicochemical properties of peptides identified in CZE and RPLC-Chip: (a) Peptide length, (b) Peptide mass, (c) Precursor m/z ratio, (d) pI, (e) Mass/length ratio, (f) GRAVY score. Statistic comparisons were performed using GraphPad Prism (La Jolla, CA, USA). (Mann-Whitney test; ***, P ≤ 0.001 and ****, P ≤ 0.0001)

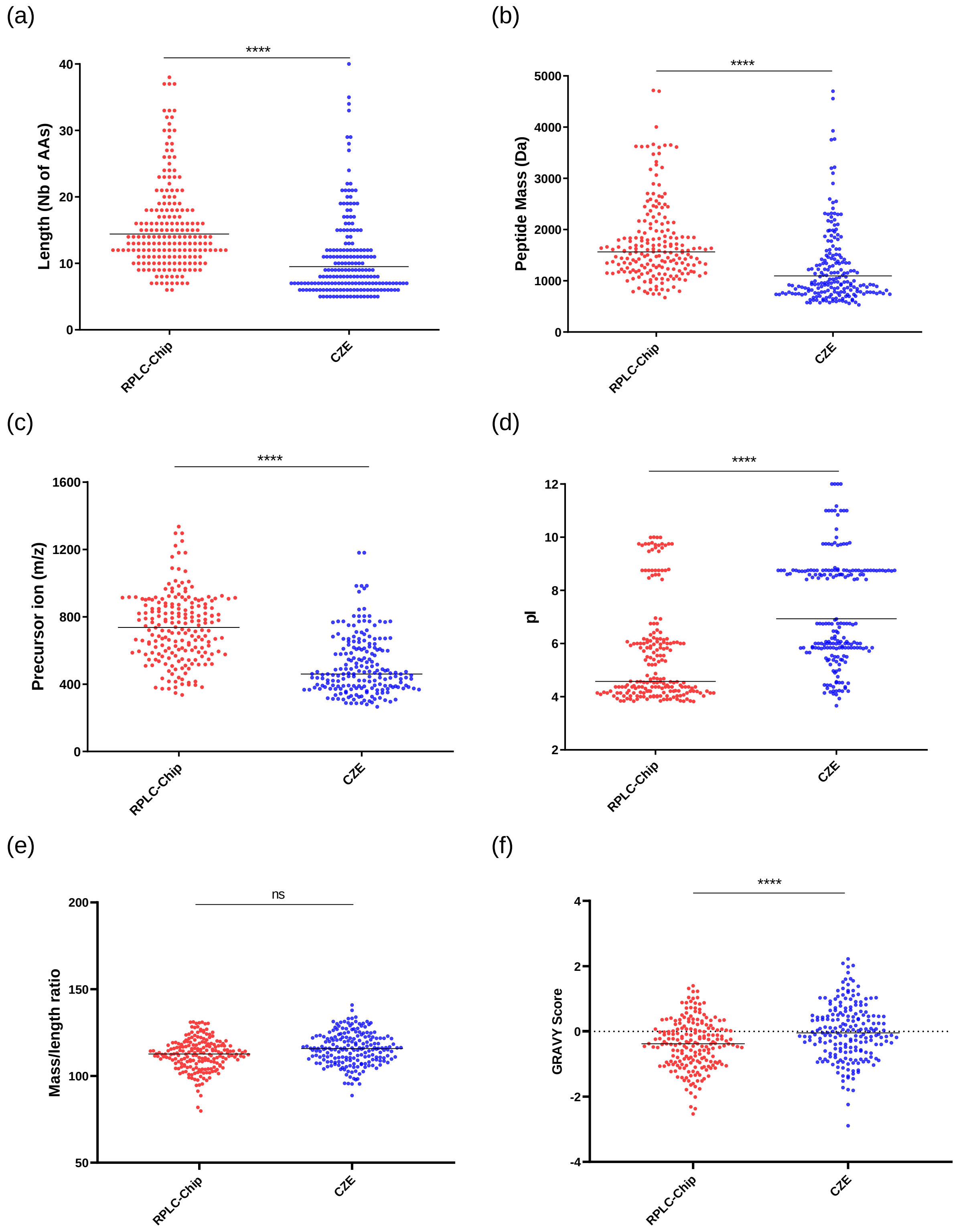

Supplement: Supplementary file 1 [file ijms-23-13350-s001.zip › ijms-1958242-supplementary.pdf]
